# Obstructive Sleep Apnea in Pregnancy: A Comprehensive Review of Maternal and Fetal Implications

**DOI:** 10.3390/neurolint16030039

**Published:** 2024-05-07

**Authors:** Antonino Maniaci, Luigi La Via, Basilio Pecorino, Benito Chiofalo, Giuseppe Scibilia, Salvatore Lavalle, Paolo Scollo

**Affiliations:** 1 Faculty of Medicine and Surgery, University of Enna “Kore”, 94100 Enna, Italy; antonino.maniaci@unikore.it (A.M.); basilio.pecorino@unikore.it (B.P.); benito.chiofalo@unikore.it (B.C.); salvatore.lavalle@unikore.it (S.L.); paolo.scollo@unikore.it (P.S.); 2 Anesthesia and Intensive Care Department, Policlinico “G.Rodolico—San Marco” Hospital, 95123 Catania, Italy; 3 Gynecology and Obstetrics Department, Giovanni Paolo II Hospital, ASP 7, 97100 Ragusa, Italy; giuseppe.scibilia@asp.rg.it

**Keywords:** obstructive sleep apnea, pregnancy outcomes, continuous positive airway pressure, gestational hypertension, prenatal health

## Abstract

Obstructive sleep apnea (OSA) is a prevalent yet underdiagnosed condition in pregnancy, associated with various maternal and fetal complications. This review synthesizes the current evidence on the epidemiology, pathophysiology, and neurological consequences of OSA in pregnancy, along with the potential management strategies. Articles were sourced from the PubMed, EMBASE, and Cochrane databases until 2023. Our comprehensive review highlights that the incidence of OSA increases during pregnancy due to physiological changes such as weight gain and hormonal fluctuations. OSA in pregnancy is linked with gestational hypertension, pre-eclampsia, gestational diabetes, and potential adverse fetal outcomes such as intrauterine growth restriction and preterm birth. Continuous positive airway pressure (CPAP) therapy remains the most effective management strategy for pregnant women with OSA. However, adherence to CPAP therapy is often suboptimal. This comprehensive review underscores the importance of the early recognition, timely diagnosis, and effective management of OSA in pregnancy to improve both maternal and fetal outcomes. Future research should focus on enhancing screening strategies and improving adherence to CPAP therapy in this population.

## 1. Introduction

Obstructive sleep apnea (OSA) is a prevalent condition characterized by repeated episodes of partial or complete upper airway obstruction during sleep, leading to intermittent hypoxia and fragmented sleep [1,2,3,4,5]. While the general population is at risk, pregnant women are particularly susceptible due to physiological changes that occur during pregnancy, such as weight gain and edema that can exacerbate airway collapsibility, as well as hormonal fluctuations that may affect respiratory drive and muscle tone [6,7]. In addition, the growing fetus and the associated increase in body mass can lead to mechanical pressure on the respiratory system [8,9]. The enlarging uterus elevates the diaphragm, thereby reducing lung capacity and increasing the effort of breathing [10]. This can contribute to a higher likelihood of upper airway collapse during sleep when muscle tone naturally decreases. This comprehensive review aims to synthesize the current knowledge of OSA’s impacts on maternal and fetal health during pregnancy. We will explore the association between OSA, mood disorders, and cognitive dysfunction in pregnant women, along with the potential neurological outcomes for the fetus. Understanding these complex interactions is critical for developing targeted interventions and management strategies to improve the health outcomes of both mother and child.

## 2. Materials and Methods

To conduct a thorough literature review, we searched the PubMed, EMBASE, and Cochrane databases for articles published up to April 2023. The search strategy included a combination of Medical Subject Headings (MeSHs) and free-text terms to capture the concepts of “obstructive sleep apnea” and “pregnancy”. Search terms included “obstructive sleep apnea”, “sleep disordered breathing”, “pregnancy”, “gestational sleep apnea”, “preeclampsia”, “gestational hypertension”, “gestational diabetes”, “intrauterine growth restriction”, “preterm birth”, and “CPAP therapy”. Filters were applied to only include articles in English and studies involving human subjects. Studies were included if they reported on the epidemiology, pathophysiology, maternal or fetal outcomes, or management of OSA during pregnancy. We considered observational studies, randomized controlled trials (RCTs), case–control studies, cohort studies, and cross-sectional studies. Reviews, meta-analyses, and clinical guidelines were also included to provide context and discuss current recommendations. Case reports, editorials, commentaries, and studies not specifically addressing OSA in pregnancy were excluded from the review. Two reviewers independently screened titles and abstracts for relevance, followed by a full-text review to determine eligibility. Discrepancies were resolved through discussion or consultation with a third reviewer. The data extracted from the selected studies included author(s), the year of publication, study design, sample size, participant characteristics, diagnostic criteria for OSA, outcomes measured, and key findings related to OSA in pregnancy.

## 3. Prevalence and Diagnosis of OSA in Pregnancy

The prevalence of OSA in pregnant women is higher than previously recognized, with estimates varying widely depending on the population studied and the diagnostic criteria used. Studies reported prevalence rates ranging from 3.6% to 27% [11,12,13]. The variability in prevalence may be influenced by factors such as obesity, age, and the presence of other comorbidities. A large prospective cohort study was performed on 3702 pregnant women at low–normal risk of OSA. This study collected data using a four-channel portable in-home sleep device in both early (6–15 weeks) and mid- (22–31 weeks) gestation. Portable sleep devices, also referred to as home sleep apnea tests, lack EEG and, therefore, any objective measure of sleep [14]. However, they provide data on photoplethysmography, nasal and oral airflow measures, and respiratory efforts. In this population, the prevalence of OSA was 3.6% in early pregnancy, as compared to 8.3% in mid-pregnancy [15]. Among patients diagnosed with OSA, 3% in early pregnancy had moderate to severe OSA (with an AHI of 3% > 15/h) versus 8% in mid-pregnancy. It is important to note that this study comprised a healthy population with 51% of subjects with a pre-pregnancy BMI < 25 kg/m^2^. Moreover, in women with a pre-pregnancy BMI > 30 (24% of study subjects), 10% were diagnosed with OSA in early pregnancy, and 10% of those had moderate to severe OSA. In another study of 128 pregnant women at high risk with a BMI > 30 and one or more of the comorbid risk factors such as chronic hypertension, pre-GDM, prior pre-eclampsia, or twin gestation, 30% of women in early (6–20 weeks), and 47% in late (28–37 weeks) pregnancy met the criteria for sleep apnea (AHI > 5/h) [16]. These data suggest that OSA becomes more common as pregnancy progresses, with the highest prevalence observed in the third trimester [17].

There is a lack of consensus on screening and diagnostic procedures for OSA in pregnant women. While some studies advocated for the routine screening of all pregnant women, especially those with high-risk factors such as obesity or hypertension, others recommended a more targeted approach [12]. 

## 4. Pathogenesis and Peculiarity of OSA in Pregnancy

Molecular mechanisms collectively contribute to a higher propensity for sleep-disordered breathing in pregnant women [18]. The hormonal, structural, and inflammatory changes that accompany pregnancy create a perfect storm for the onset or exacerbation of OSA [13]. Pregnancy is characterized by significant hormonal fluctuations that can exacerbate the risk of developing OSA [19,20]. Progesterone levels rise, which, while beneficial for maintaining the uterine lining and supporting the pregnancy, can also lead to increased upper airway collapsibility [21]. Progesterone is a respiratory stimulant that increases the sensitivity of the brain’s carbon dioxide monitoring system and can cause an increase in the overall drive to breathe, potentially leading to more forceful attempts to overcome any upper airway resistance [22,23]. This might paradoxically contribute to more episodes of airway collapse during sleep [13]. Estrogen levels also increase during pregnancy, which can contribute to the swelling of the mucous membranes lining the nose and oropharynx, thereby narrowing the airway and increasing resistance to airflow during respiration, probably being a direct contributor to the mechanical obstruction characteristic of OSA. Moreover, the deposition of fat in the soft tissues of the neck and the increase in breast size can put additional pressure on the upper airway. These anatomical changes can contribute to the narrowing of the airway and the increasing airway resistance, predisposing women to the development of OSA [23]. Pregnancy is also associated with increased blood volume and fluid retention [24]. This excess fluid can accumulate in the tissues of the neck and the peripharyngeal space, further narrowing the airway and increasing the risk of obstruction during sleep. Fluid retention is particularly pronounced in the third trimester and in the presence of pregnancy-related hypertensive disorders, which can exacerbate OSA severity [24]. 

Systemic inflammation increases during pregnancy as part of the normal immunological adaptation to support the developing fetus [21,25,26]. However, this heightened inflammatory state can also contribute to airway inflammation and swelling, which can increase the risk of OSA [25,26]. Pregnant women often experience insulin resistance and metabolic changes, which can be associated with weight gain and increased fat deposition, including in the neck region, contributing to a narrowed airway [27,28]. There may also be genetic and epigenetic factors that predispose pregnant women to OSA [29]. These could include variations in genes related to inflammation, metabolism, and adipose distribution, which might be modulated differently during pregnancy, affecting airway patency and respiratory control mechanisms [29]. The molecular mechanisms through which OSA affects maternal and fetal health are multifaceted. Intermittent hypoxia—the hallmark feature of OSA—can invoke a systemic inflammatory response, characterized by the release of pro-inflammatory cytokines, oxidative stress, and endothelial dysfunction, potentially affecting placental function and fetal development [30,31,32]. Additionally, the sleep fragmentation associated with OSA may lead to alterations in maternal hormonal profiles, which can have downstream effects on mood regulation and cognitive function [9,10]. Furthermore, the compounding effects of sleep disruption and hypoxia may precipitate or exacerbate mood disorders, such as depression and anxiety [17,33], which are already more prevalent during pregnancy [34]. Cognitive disturbances, including impairments in memory and executive function, may also arise as a consequence of OSA during pregnancy, posing potential long-term impacts on maternal health [12,35]. Moreover, there is a growing body of evidence suggesting that maternal OSA can have direct neurological implications for the fetus. The intermittent hypoxic environment may influence fetal brain development and lead to neurobehavioral disorders in offspring [32], raising questions about the long-term neurodevelopmental trajectory of children born to mothers with untreated OSA [31].

## 5. Associations between OSA and Maternal Mood Disorders

The associations between OSA and maternal mood disorders are complex and bidirectional, rooted in both physiological disruptions and psychological stressors. OSA induces intermittent hypoxia and fragmented sleep, which can lead to significant alterations in neurochemical regulation and hormonal balance [36]. This dysregulation can result in heightened stress responses and exacerbate susceptibility to mood disorders such as depression, post-traumatic stress disorder (PTSD), and anxiety [37,38,39]. 

From a pathophysiological standpoint, the chronic sleep disruption experienced by those with OSA impairs the restorative processes of sleep, leading to increased levels of cortisol, the stress hormone, which has been implicated in mood disturbances [40]. In addition, the repetitive oxygen desaturation and reoxygenation cycles characteristic of OSA can generate oxidative stress and inflammatory responses, further contributing to neurological changes and mood dysregulation [41]. A cross-sectional study was recently conducted by Rubio et al. [42] on pregnant women with sleep disorders. In this paper, approximately 29% of women had poor sleep quality, and 6.2% were at high risk for OSA. The prevalence of psychiatric symptoms was high in this cohort with 25.1%, 32.5%, and 30.9% of women reporting symptoms of antepartum depression, antepartum anxiety, and PTSD, respectively. Women with a high risk of OSA had higher odds of antepartum depression, generalized, and PTSD symptoms as compared with those with a low risk of sleep apnea. Moreover, the psychological impact of OSA, including chronic fatigue and the stress of coping with a sleep disorder, can also weigh heavily on mental well-being, potentially triggering feelings of inadequacy or frustration that can spiral into more severe mood disorders [38]. 

For pregnant women already experiencing hormonal changes and the emotional demands of pending motherhood, the added strain of OSA may significantly heighten the risk of developing mood disorders. Moreover, pregnancy itself is a period of increased vulnerability to mood disturbances due to fluctuating estrogen and progesterone levels, which can influence neurotransmitter systems associated with mood regulation [17,37]. When compounded with OSA, the hormonal imbalances may become more pronounced, leading to a greater incidence and severity of mood disorders in pregnant women, particularly when cancer is involved [43].

## 6. OSA-un-Related Sleep Disorders in Pregnancy

Obtaining sufficient sleep quality and quantity in addition to assessing and treating sleep disorders should be a priority for pregnant women and their providers [44]. Among possible sleep disorders, besides OSA, recent studies have found that 38.2% of pregnant women experience insomnia and 20% experience restless leg syndrome [45,46].

### 6.1. Insomnia

Since a lower quality of sleep is typical throughout a healthy pregnancy, pregnant women are more susceptible to developing clinical insomnia. According to a recent meta-analysis and several studies, 38.2% of pregnant women report having sleeplessness, and the prevalence of insomnia rises with the length of pregnancy [46,47,48,49]. However, it is challenging to distinguish between normative and clinical sleep complaints due to high base rates of sleep disturbance. Clinical insomnia is defined by the DSM-5 as problems in falling asleep, remaining asleep, or waking up too early on at least three nights per week for at least three months, accompanied by deficits in daytime functioning. Because the symptom changes occur over a very short period of time, it might be difficult to determine clinical levels of insomnia in this population, especially when it comes to meeting the 3-month duration requirement. The evidence indicates that inadequate sleep is predictive of problems for both the mother and the fetus, regardless of how sleep complaints are classified. OSA-related insomnia during pregnancy is linked to higher risks of hypertension, prolonged deliveries, and premature birth and/or cesarean section [50,51]. Gestational diabetes risk is also linked to shorter sleep durations during pregnancy (less than 6.25 h) and later sleep onset [52]. A plausible explanation for these heightened risks could be heightened inflammatory reactions brought on via insufficient sleep, which intensifies the prenatally existing elevated inflammatory state. In late pregnancy, there is a correlation between higher levels of inflammatory biomarkers and lighter and less slow-wave (deep) sleep [53].

### 6.2. Restless Leg Syndrome (RLS)

RLS is 20% more common during pregnancy, according to a recent epidemiological study [54]. Women over 35 and those who report other sleep problems are at higher risk. New-onset RLS affects one in five pregnant women; the incidence rises with increasing parity, gestational age, maternal age, and maternal obesity [55]. Fortunately, RLS symptoms resolve quickly after delivery for up to 80% of women [56]. Although the exact causes of RLS in pregnancy are unknown, theories include hormonal changes (such as prolactin, progesterone, and estrogen), metabolic abnormalities (such as iron and folate insufficiency), psychological problems, and stress in pregnant women [57]. Although few research studies have looked at the incidence of periodic limb movements in pregnancy, those that have suggested that there is minimal evidence for a clinically significant increase. Women who experience RLS during pregnancy may also be at increased risk of developing this condition again later in life. A study by Cesnik et al. [57] found that women with a history of RLS during pregnancy had a higher prevalence of RLS in the postpartum period and beyond, compared to those without RLS during pregnancy. This suggests that pregnancy-related RLS may be a marker for an underlying predisposition to this disorder. Healthcare providers should be aware of this potential long-term risk and provide appropriate follow-up and management for women with RLS during pregnancy.

## 7. Influence of Maternal OSA on Fetal Outcomes

Maternal OSA has been associated with various adverse fetal outcomes. Several studies have investigated the relationship between maternal OSA and fetal growth restriction, preterm birth, and low birth weight. A meta-analysis by Ding et al., found that maternal OSA was associated with an increased risk of preterm birth (odds ratio [OR] = 1.75, 95% confidence interval [CI]: 1.21–2.55) and low birth weight (OR = 1.39, 95% CI: 1.14–1.65) [58]. Similarly, a systematic review by Pamidi et al., reported that maternal OSA was associated with an increased risk of preterm birth (OR = 1.94, 95% CI: 1.32–2.85) and low birth weight (OR = 1.52, 95% CI: 1.24–1.85) [59].

Fetal growth restriction has also been linked to maternal OSA. A study by Fung et al., found that pregnant women with OSA had a higher incidence of fetal growth restriction compared to those without OSA (7.1% vs. 2.6%, *p* = 0.004) [60]. Additionally, a prospective study by Kneitel et al., reported that maternal OSA was associated with decreased fetal growth velocity and a higher risk of small for gestational age (SGA) infants (OR = 2.96, 95% CI: 1.02–8.62) [61].

**Figure 1:**
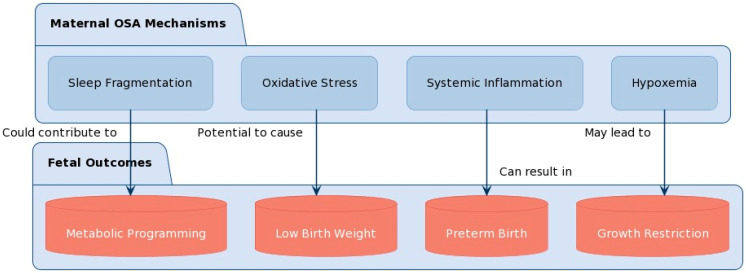
Pathophysiological mechanisms contributing to fetal outcomes.

The mechanisms underlying the association between maternal OSA, and adverse fetal outcomes are not fully understood. However, several potential pathways have been proposed (Figure 1). Intermittent hypoxia and sleep fragmentation associated with OSA may lead to oxidative stress, inflammation, and endothelial dysfunction, which can adversely affect placental function and fetal development [62]. Additionally, OSA-induced sympathetic activation and hormonal changes may contribute to altered fetal growth and development [63].

It is important to note that the majority of studies investigating the impact of maternal OSA on fetal outcomes have been observational in nature, and the evidence is not always consistent. Some studies have found no significant association between maternal OSA and adverse fetal outcomes [64,65]. These discrepancies may be due to differences in study populations, diagnostic criteria for OSA, and confounding factors. Further well-designed prospective studies are needed to better understand the relationship between maternal OSA and fetal outcomes and to develop effective strategies for screening and managing OSA during pregnancy.

## 8. Effectiveness of OSA Treatment on Maternal and Fetal Health

The treatment of OSA during pregnancy has profound implications for both maternal and fetal health [44,64]. Research has consistently shown that untreated OSA in expectant mothers can lead to a spectrum of complications, such as hypertension, pre-eclampsia, gestational diabetes, and adverse neonatal outcomes like low birth weight and preterm birth. 

Several studies have investigated the impact of OSA on pregnancy outcomes. A meta-analysis by Ding et al. [58] found that maternal OSA was associated with a significantly increased risk of gestational diabetes mellitus (OR 1.89), pre-eclampsia (OR 1.58), and preterm birth (OR 1.36). Another study by Louis et al. [64] reported that OSA during pregnancy was linked to a higher incidence of low-birth-weight infants and neonatal intensive care unit admissions. The mechanisms underlying these adverse outcomes are multifaceted. Intermittent hypoxia, a hallmark of OSA, can lead to oxidative stress and systemic inflammation, which may impair placental function and fetal growth [65]. Additionally, the sympathetic activation and endothelial dysfunction associated with OSA can contribute to the development of gestational hypertensive disorders [66].

However, when OSA is addressed, particularly through the use of continuous positive airway pressure (CPAP) therapy, these risks are significantly diminished [67]. CPAP therapy serves as the mainstay treatment for OSA and operates by delivering a stream of pressurized air via a mask to maintain open airways during sleep, addressing the apneic episodes and the resultant hypoxemia and sleep fragmentation characteristic of OSA [68,69]. The therapeutic effects of CPAP extend beyond the mere alleviation of obstructed breathing; they bring about a cascade of beneficial molecular changes within the body [70,71,72,73,74]. With consistent use of CPAP, there is a marked reduction in systemic inflammation [75]. Levels of pro-inflammatory cytokines, such as TNF-α and IL-6, are lowered, which is beneficial not only for the mother’s health but also for the fetal environment [44]. Furthermore, the hormonal disturbances often seen in OSA, including elevated cortisol and catecholamine levels that can disrupt fetal development, are stabilized [76]. Improved maternal oxygenation through CPAP use also diminishes oxidative stress, thus fostering better endothelial function [77,78]. A prospective study by Guilleminault et al. [79] found that CPAP treatment in pregnant women with OSA led to a significant reduction in pre-eclampsia (from 19% to 7%) and gestational diabetes (from 31% to 10%) compared to untreated controls. Similarly, a randomized controlled trial by Poyares et al. [80] demonstrated that CPAP therapy in pregnant women with OSA improved blood pressure control and reduced the risk of preterm delivery. Another significant molecular change with CPAP treatment is the improvement in metabolic regulation [72]. This encompasses enhanced glucose metabolism and insulin sensitivity, which are particularly crucial in reducing the risk of gestational diabetes—a condition with long-term health implications for both mother and child [72]. Additionally, the regulation of appetite-controlling hormones through better sleep quality may aid in maintaining a healthy weight during pregnancy.

A recent systematic review by Nugent et al. [81] was conducted in order to determine whether treating pregnant women with OSA using CPAP would improve maternal or fetal outcomes, as compared with no treatment or delayed treatment. In this review including seven clinical trials, very low-quality evidence suggests that CPAP may reduce BP and the risk of pre-eclampsia, reduce prematurity, and increase birth weight. Despite these clear benefits, adherence to CPAP therapy remains a challenge [68], particularly during pregnancy, when comfort with the treatment apparatus is of increased importance. Nonetheless, the evidence supports the effectiveness of CPAP in not only improving sleep quality and reducing the frequency of apneic events but also in creating a healthier intrauterine environment for the fetus [52]. However, more research is warranted on this topic to draw further conclusions. In this context, two RCTs have completed their study and are in the process of publishing their findings. The study by Dominguez et al. [82] is a multicenter RCT investigating the impact of CPAP on the incidence of pre-eclampsia in high-risk pregnant women with OSA. Another ongoing multicentric RCT, the SAFER (Sleep Apnea during Pregnancy: Evaluation of Risks) study [83], is currently aiming at recruiting 1500 patients to provide conclusive results on the effect of CPAP in treating OSA in pregnancy. These studies underscore the importance of screening for and treating OSA during pregnancy to optimize both maternal and fetal health outcomes. As more high-quality evidence emerges, the integration of OSA management into routine prenatal care may become increasingly crucial.

## 9. Discussion

This comprehensive review synthesizes current research on OSA during pregnancy, a condition increasingly recognized but often underdiagnosed. Studies report a prevalence rate ranging from 3.6% to 27%, suggesting OSA is more common in pregnant women than previously believed. The review identifies several physiological changes during pregnancy that contribute to the onset or worsening of OSA. These include weight gain, upper airway narrowing due to edema, increased progesterone levels that relax airway muscles and enhance collapsibility, and the upward displacement of the diaphragm by the growing uterus. These factors together create a conducive environment for OSA development. Furthermore, the review delves into the consequences of OSA on maternal health, particularly its association with mood disorders like anxiety and depression. The sleep disruption and intermittent hypoxia caused by OSA can alter neurotransmitters, hormones, and inflammatory markers, potentially increasing susceptibility to mood disturbances. This is especially concerning as pregnancy is already a period prone to mood disorders due to hormonal changes. The review also explores the potential adverse effects of maternal OSA on fetal outcomes and neurodevelopment, linking OSA to complications such as intrauterine growth restriction, pre-eclampsia, preterm birth, and low birth weight. While intermittent hypoxemia and inflammatory responses are suspected contributors, the exact mechanisms remain unclear, and more detailed longitudinal studies are necessary. Regarding treatment, CPAP is affirmed as the standard effective intervention for improving sleep quality and health outcomes for both mother and fetus. However, adherence to CPAP therapy presents significant challenges. The review points out the scarcity of high-quality randomized controlled trials comparing CPAP with other treatments like oral appliances or lifestyle changes and calls for more research to optimize OSA management strategies in pregnancy and strengthen the basis for clinical guidelines.

## 10. Future Directions

The physiological changes of pregnancy, including weight gain, edema, and hormonal fluctuations, predispose pregnant women to OSA onset or exacerbation. Maternal OSA is associated with increased risks of gestational hypertension, pre-eclampsia, gestational diabetes, preterm birth, and adverse fetal outcomes. These complications underscore the importance of early recognition and timely OSA diagnosis and management in pregnant women. CPAP therapy remains the most effective treatment for OSA in pregnancy, leading to marked improvements in sleep quality and maternal–fetal health outcomes [52]. However, adherence to CPAP is suboptimal and requires additional support [57]. There is a need for large-scale longitudinal studies with robust methodologies to better characterize OSA severity and prevalence across all trimesters of pregnancy. Larger randomized controlled trials are warranted to compare CPAP with other potential OSA treatments in pregnant women and definitively establish optimal management strategies. Future research should focus on developing and validating OSA screening tools, specifically for pregnant populations, to enable early diagnosis and treatment initiation. Additional studies are needed to identify predictors of poor CPAP adherence and explore targeted interventions to improve compliance. Developing comfortable CPAP interfaces and customized therapeutic support programs tailored for expectant mothers may enhance adherence. Finally, long-term studies tracking neurodevelopmental outcomes in children born to mothers with OSA are crucial for determining the lasting impacts of prenatal sleep apnea exposure. This collective research will provide the foundation for evidence-based guidelines to optimize maternal and fetal well-being in pregnancies complicated by OSA. Moreover, like in the gynecological oncology field, the multidisciplinary approach is the mandatory approach to manage these patients in order to improve maternal and fetal outcomes [43].

## 11. Conclusions

The review underscores the heightened prevalence and complex etiology of OSA in pregnancy, linking it to physiological changes and increased risks of maternal and fetal complications. It highlights the bidirectional relationship between OSA and mood disorders in pregnant women, exacerbated by sleep disruption and hormonal imbalances. CPAP therapy is affirmed as the most effective treatment for OSA, despite challenges with patient adherence. The review calls for increased vigilance in screening and managing OSA in pregnant women, emphasizing a multidisciplinary approach. There is a clear need for more research to optimize OSA treatment strategies and develop targeted interventions to ensure adherence. Long-term studies are also necessary to fully understand the impacts of prenatal OSA on child development.

## Data Availability

No new data were created or analyzed in this study. Data sharing is not applicable to this article.
